# Is There a Correlation Between Dog Obesity and Human Obesity? Preliminary Findings of Overweight Status Among Dog Owners and Their Dogs

**DOI:** 10.3389/fvets.2021.654617

**Published:** 2021-07-09

**Authors:** Deborah E. Linder, Sasha Santiago, Eli D. Halbreich

**Affiliations:** ^1^Tufts Institute for Human-Animal Interaction, Tufts University, Grafton, MA, United States; ^2^Department of Clinical Sciences, Cummings School of Veterinary Medicine at Tufts University, North Grafton, MA, United States; ^3^Department of Psychology, Tufts University School of Arts and Sciences, Medford, MA, United States

**Keywords:** obesity, obesity prevention, One Health, human-animal interaction, dogs, human-animal bond

## Abstract

**Background and Aim:** Obesity is a serious health issue in people and their pets, with a need for innovative and engaging prevention strategies. One possible strategy is a One Health approach incorporating dogs into prevention programs; however, little data exist in the U.S. about the association between weight status among dog owners and their dogs. The objective of this study was to determine if there was an association between body mass index of adult dog owners and corresponding weight status in their dogs.

**Materials and Methods:** This cross-sectional correlation study collected data from 38 adult dog owners aged 18 years and older and their dogs at three pet festivals throughout New England. Body mass index of dog owners and body condition scores of dogs were measured on site. Spearman correlation was used to compare weight status in dogs and their owners.

**Results:** The median body mass index of dog owners was 26 (range of 17–53) and the median body condition score of dogs was 6 (range of 4–9). Frequency of overweight and obesity in dog owners was 31.6 and 26.3%, respectively, and 50.0 and 13.2% in dogs, also, respectively. Owner body mass index was positively correlated with dog body condition score (*r* = 0.60, *p* < 0.001).

**Conclusion:** Our findings support a possible association between overweight status in dogs and their owners. These findings could be leveraged in future interventions to promote healthier and more active lifestyles for both dog owners and their dogs in an engaging and innovative obesity prevention approach.

## Introduction

The obesity epidemic is a prominent health concern affecting adults and children in the United States, and since the 1970s its prevalence has been increasing at an alarming rate (1–4). The prevalence of obesity has consistently risen since 1999 and it is predicted that by 2030 78% of American adults will be either obese or overweight, with nearly 50% of adults being obese (4). Furthermore, it is predicted that 33% of children and 50% of adolescents will be either overweight or obese by 2030 (4). In parallel to human obesity, pet obesity is also a serious and growing concern. In 1995, 34.1% of adult dogs in the United States were overweight or obese (5). More recent studies estimate that the prevalence of overweight and obesity in dogs has increased to over 50% of the canine population (6).

With such concerning statistics, there is a need for engaging, innovative, and effective strategies to not only manage, but even more importantly prevent obesity in the US population. One emerging consideration for obesity prevention strategy is the incorporation of companion pets to promote engagement in healthier behaviors. There are many similarities between dog and human obesity, including clinical consequences of obesity, the needs for nutrition and physical activity behavior change, understanding physical vs. emotional hunger, and the influence of media on food selection (7, 8). Risk factors for obesity are also similar between dog owners and their dogs, such as sedentary lifestyles (9).

Given these parallels and the potential impact of the human-animal bond on healthy behaviors, more information is needed on the shared health implications of obesity in people and their pets. A study done by Nijland et al. (10) in the Netherlands found that overweight in adult owners is correlated with overweight in their pet dogs. These findings reinforce the implication that owners may apply their own health choices, specifically those regarding food and exercise, to their dogs (10). Another study done by Delicano et al. (9) found an association between owning a dog with a diagnosis of diabetes and type 2 diabetes in the owner among over 200,000 owner-dog pairs in Sweden. The authors hypothesized that shared health behaviors (e.g., physical activity) and shared environmental exposures could explain the correlation (9). Findings such as these have also prompted whether weight status of owners and their pets might impact weight bias among veterinarians. For example, Pearl et al. (11) found that both veterinarians and veterinary students respond and act differently toward overweight dogs and their owners compared to lean dogs and their owners. The researchers recommended further research into the impact of weight bias on clinical decision making and interactions with pet owners, specifically calling for study into the effects of respectful and non-stigmatizing language related to the weight of pets and their owners (11). Due to the strong bond and shared health behaviors between people and their dogs, one novel approach to addressing the obesity epidemic is incorporating dogs into weight loss interventions for shared “One Health” benefits, where both the dog and the owner can increase healthy behaviors.

While a number of studies have been published considering this One Health approach of using the human-animal bond to facilitate weight loss in both humans and their dogs [i.e., (12–15)], additional information could prove useful to build on these studies to better inform future successful interventions. For example, while the People and Pets Exercising Together (PPET) study (14) included pairs of dog owners and dogs in the US that were both obese, these participants were specifically recruited for this study, so it is not known how frequent this correlation is within the general US population. While there was a correlation found in Europe, it is not known if dog owners and their dogs in the United States share a similar association in body composition and obesity. Therefore, the objective of this study was to determine if there is a correlation between the body composition of dogs and the body composition of their owners. Additional information on this possible correlation in the US would build on current knowledge and allow for more informed and better designed future intervention programs that promote healthier and more active lifestyles for both people and their dogs.

## Methods

### Participants

Data were collected at three pet festivals at different locations throughout Central New England. The festivals were open to the general public and their pets. All festivals were community-based, included fundraising components (e.g., optional raffles, etc.) for various animal organizations, and provided family style entertainment such as food vendors, informational booths on pet and/or human health, and pet friendly activities (e.g., dog trick or costume competition). Visitors over the age of 18 with their dog present were asked to participate in a study on dog and owner health as they walked by an educational booth on pet health staffed by the study investigators. Thirty-eight owners and their dogs participated in the study anonymously and no identifying information was collected. Inclusion criteria for dog owners required that they were at least 18 years of age, were the primary owner of the dog, and were fluent in English in order to provide informed consent. Inclusion criteria for animals included dogs for which body condition score (BCS) measurement is validated (i.e., over 1 year of age and not currently in a state of gestation or lactation). Prior to recruitment and study commencement, this study was reviewed by both the Tufts University Institutional Review Board and Institutional Animal Care and Use Committee.

### Measurements

Dog owner body composition was determined by measuring height and weight to calculate body mass index (BMI) as described in Nijland et al. (10). BMI was measured in kg/m^2^ and defined as underweight (<18.5), healthy range (18.5–24.9), and overweight (25–29.9) and obese [>30; (16)]. A stadiometer was used to measure participant height. Weight was collected using a scale with the weight output covered by a shield to keep the weight of the participant private. Participants were told their weight if requested. Demographic information was not collected or asked of participants.

Body composition of dogs was determined by measuring BCS, in which the primary investigator visually inspected and palpated the fat tissue over the sides of the dog (17). BCS was measured on a scale from 1 (underweight) to 9 (obese) and defined as underweight (1–3), healthy range (4–5), overweight (6–7), and obese [8–9; (18)].

### Data Analysis

Data were assessed for normality. Both BMI and BCS were skewed and thus reported as median (range). Spearman's correlation was used to analyze the association between BMI of owners and BCS of dogs. A sensitivity analysis was performed by excluding one outlier (participant with a high BMI) to determine robustness. Chi-square test was used to analyze the difference between categories of weight status in dogs and dog owners. Commercial statistical software (IBM Corp. Released 2013. IBM SPSS Statistics for Windows, Version 22.0. Armonk, NY: IBM Corp.) was used for all analyses, and a *P* value < 0.05 was considered significant.

## Results

**Figure 1:**
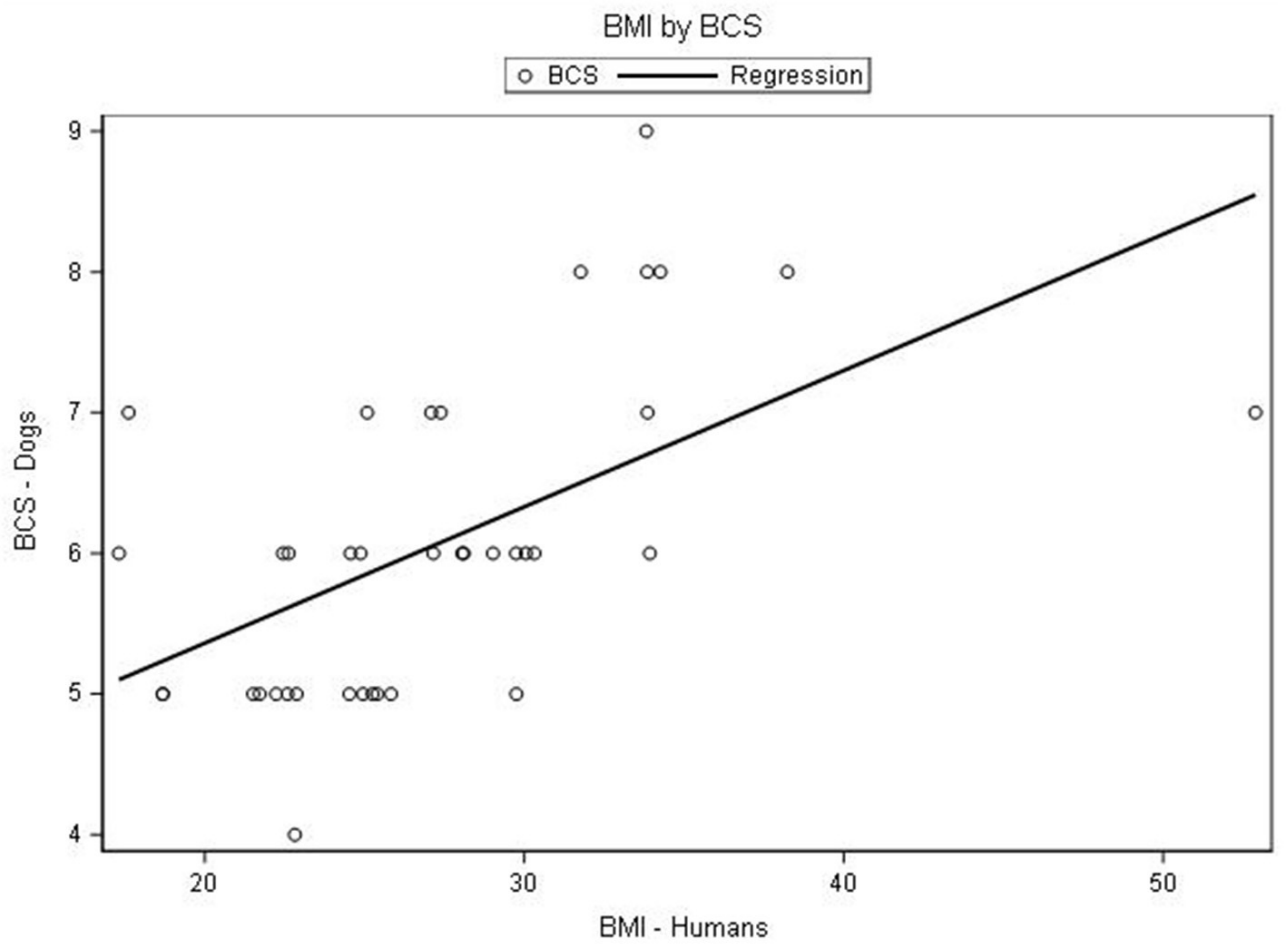
Scatterplot showing distribution of body mass index (BMI) of 38 dog owners and body condition score (BCS) of their dog. *BMI was measured in kg/m^2^ and defined as underweight (<18.5), healthy range (18.5–24.9), and overweight (25–29.9) and obese (>30). ^†^BCS was measured on a scale from 1 (underweight) to 9 (obese) and defined as underweight (1–3), healthy range (4–5), overweight (6–7), and obese (8–9).

The median BMI of dog owners was 26 (range of 17–53) while the median BCS of dogs was 6 (range of 4–9). Body composition as defined by BMI in dog owners and BCS in dogs, as well as weight status, is described in Table 1. The distribution of BMI in dog owners compared to BCS of their dogs is shown as a scatterplot in Figure 1.

**Table 1:** BMI of dog owners and BCS of dog study participants presented in median, range, and weight status.

	***N***	**Median**	**Range**
BMI (kg/m^2^)^*^	38	26	17.3–52.9
Healthy (*n*, %)	16 (42.1%)		
Overweight (*n*, %)	12 (31.6%)		
Obese (*n*, %)	10 (26.3%)		
BCS^†^	38	6.0	4.0–9.0
Healthy (*n*, %)	14 (36.8%)		
Overweight (*n*, %)	19 (50.0%)		
Obese (*n*, %)	5 (13.2%)		

*N, number of participants; BMI, body mass index (kg/m^2^); BCS, body condition score*.

* *BCS was measured on a scale from 1 (underweight) to 9 (obese) and defined as underweight (1–3), healthy range (4–5), overweight (6–7), and obese (8–9)*.

† *BMI is defined as underweight (<18.5), healthy range (18.5–24.9), and overweight (25–29.9) and obese (>30)*.

Frequency of overweight (BMI between 25 and 29.9) of dog owners was 31.6% and 50% (BCS 6–7) in dogs (Table 1). Obesity (BMI >30) of dog owners was 26.3 and 13.2% (BCS 8–9) in dogs (Table 1). A Chi-Square test on the weight categories of healthy, overweight, and obese among dogs and dog owners further showed a significant relationship between weight status of dogs and their owners (*p* = 0.0002).

Spearman's correlation demonstrated that the BMI of the dog owners and the BCS of their dogs had a strong, positive correlation (*r* = 0.60, *P* < 0.001). A sensitivity analysis was performed by excluding one outlier (participant with high BMI) and robustness was confirmed with a strong statistically significant correlation with both primary and sensitivity analysis.

## Discussion

The present findings support an association between the BMI of dog owners and the BCS of their dogs in New England. These findings are similar to a previous study by Nijland et al. (10), which showed correlation (*r* = 0.31) in Europe between the degree of overweight of dog owners and their dogs. In the previous study, there were 47 pairs of owners and dogs, and the owners had to have owned the dogs for at least 1 year to meet inclusion criteria (10). In contrast to the results of the current study and Nijland and colleagues' study, the results of a questionnaire study distributed across 11 European countries to over 3,000 dog owners indicated that there was no direct correlation between obesity in owners and their dogs (19). However, this discrepancy could be explained by the difference in study methodology (i.e., self-report questionnaire vs. in-person study) or by the owners' ability to accurately assess the body condition of their dog. Findings from both the current study and Nijland and colleagues' study indicate that there is a possible and likely correlation between the body composition of dogs and their owners.

For broader implications, these findings provide evidence for the presence of shared health concerns between dog owners and their dogs. Because the obesity epidemic similarly affects both people and pets, further information about these shared health concerns could lead to a higher rate of adoption and funding for innovative treatment and prevention approaches in the future. Obesity further impacts public health through the co-morbid conditions that affect both people and dogs such as diabetes and osteoarthritis (7, 8). As noted in the study by Delicano et al. (9), their findings of shared risk of diabetes for dog owners and their dogs may point to possible shared diabetogenic health behaviors or environmental exposures as a possible explanation for these shared health concerns. It will be critical in future studies to further investigate the potential mechanism(s) behind shared health concerns beyond behaviors such as overeating and lack of exercise, but also consider cross-species implications of misinformation, health behaviors, psychosocial impact of the human-animal bond or environmental exposures, etc. Given these shared health concerns, future studies could also investigate how to more effectively leverage the dog-owner relationship to promote healthier and more active lifestyles for both dogs and owners. As mentioned above, a number of studies have already begun investigating this relationship [i.e., (12, 13, 15)], but there are a number of additional directions for research, including cross-cultural considerations and large-scale studies. The preliminary findings from this limited population could build on previous knowledge to better develop and inform study design of future large-scale studies (for example, informing the potential target population and recruitment considerations).

A prior study performed in Chicago was the first to demonstrate feasibility of a dog-owner weight loss program (14). Participants reported that their dogs were a constant source of support, motivation, and enjoyment throughout the program (14). Over the course of the 12-month program, participants paired with dogs reduced their weight by an average 4.7% (*SD* = 4.8) and dogs reduced their weight by an average 14.9% [*SD* = 8.8; (14)]. Owners were also motived by the sense of pride they felt as they watched their pet lose weight and look and feel better (14). Understanding the shared health implications dogs have with their owners may also impact owner motivation. A recent review of dog-walking highlights how the relationship people have with their dogs and desire to improve their dog's health can be potential motivating factors to increase walking (20). The results from this study can be incorporated into dog-owner programs in which improving dog health as an outcome may impact dog owner engagement in future interventions.

### Study Limitations

These findings also have limitations, foremost the sample size of the study. Enrollment was limited by participant willingness to have their height and weight measured in public. Though these measurements were done anonymously and with privacy, some festival attendees told investigators that they declined to participate due to this factor (though refusal rate and rationale for refusal was not asked nor documented). Another limitation is that the population of dog owners that attend pet festivals and participate in studies on body composition may not be representative of the general dog-owning population. Dog owners who prefer not to socialize with other dog owners or prefer that their dogs not socialize with other dogs would not be represented in the study. Additionally, there may have been owners who declined to participate due to concerns of embarrassment from themselves or their dogs being overweight. However, a range of dog owners and dogs participated, spanning BMI and BCS scores of healthy, overweight, and obese, which indicates our population was representative of all weight categories. Overall, however, these are preliminary findings limited to the New England dog population that warrant future studies with a larger sample to ensure a fully representative sample of the dog-owning population.

### Future Directions

Future studies could examine the possible mechanism behind this association for obesity in dog owners and dogs and what further health problems dogs and their owners might share. Additionally, motivation to improve the health of dogs as an incentive to engage in One Health weight loss programs could be further explored with dog owners, as Candellone et al. (13) explored in the case report of one family. The preliminary findings from this limited population also allow for more informed study design of future studies such as potential target populations and recruitment considerations. Given these shared health concerns, healthcare providers in veterinary and family medicine hold key positions for promoting healthy lifestyle behaviors and are well-positioned for leveraging the human-animal bond in positive ways for both people and their companion pet dogs.

## Conclusions

A significant finding of this study showed that owner body mass index was positively correlated with dog body condition score in the New England dog population. Future studies are warranted to further understand the dog-owner relationship and how it could be leveraged to improve healthy behaviors for people and their pets.

## Data Availability Statement

The raw data supporting the conclusions of this article will be made available by the authors, without undue reservation.

## Ethics Statement

The studies involving human participants were reviewed and approved by Tufts University Institutional Review Board. The ethics committee waived the requirement of written informed consent for participation. The animal study was reviewed and approved by Tufts University Institutional Animal Care and Use Committee. Written informed consent for participation was not obtained from the owners because verbal consent was obtained and a verbal script for consent was reviewed and approved through Tufts University IRB and IACUC review.

## Author Contributions

DL and SS contributed to conception and design of the study, collected data, and performed preliminary analysis. DL and EH conducted further analysis of the data and interpretation for future directions. DL, SS, and EH wrote sections of the manuscript. All authors contributed to the article and approved the submitted version.

## Conflict of Interest

The authors declare that the research was conducted in the absence of any commercial or financial relationships that could be construed as a potential conflict of interest.
